# Thigh Muscle Volume Remains Decreased on 3-Dimensional Magnetic Resonance Imaging at Minimum of 5 Years After Patellar Tendon but not Allograft Anterior Cruciate Ligament Reconstruction

**DOI:** 10.1016/j.asmr.2025.101191

**Published:** 2025-05-28

**Authors:** Collin D.R. Hunter, Natalya McNamara, Reece M. Rosenthal, Joseph Featherall, Patrick Greis, Travis G. Maak, Stephen K. Aoki, Justin J. Ernat

**Affiliations:** Department of Orthopaedics, University of Utah, Salt Lake City, Utah, U.S.A.

## Abstract

**Purpose:**

To evaluate thigh muscle volume differences in patients undergoing anterior cruciate ligament reconstruction (ACLR) with either allograft tissue or bone−patellar tendon−bone (BPTB) autografts, using 3-dimensional magnetic resonance imaging (MRI) modeling.

**Methods:**

This is a retrospective, single-center study of patients undergoing primary ACLR with either ipsilateral BPTB autograft or allograft tissue. Inclusion criteria were age 18-45 years, 5 to 8 years of follow-up, and body mass index <30. Patients were excluded if they had multiligamentous knee injuries, surgical treatment of meniscus tears (repair or reconstruction), and any subsequent knee procedures after the index ACLR. 3D MRI of the bilateral thighs was performed and images were rendered with 3D modeling software and the total thigh, anterior-, posterior-, and medial-thigh compartment musculature were individually segmented and the volumes were calculated. Data were evaluated using a *t* test, and muscle volumes were standardized as a percentage of the corresponding nonoperative extremity and then tested in comparison with the alternative graft type.

**Results:**

Ten allograft and 10 patients with BPTB were included. Patients with allograft were older at surgery (mean age 34.6 vs 23.4 years (*P* < .001) and at study MRI (mean age 40.9 vs 29.7 years; *P* < .001). Allografts showed no differences in total thigh or compartmental muscle volumes between the operative and nonoperative limbs. BPTB showed a statistically significant reduction in total thigh volume on the operative side (103 cm^3^, 97.0%, *P* = .013), whereas compartment volumes remained similar between limbs. Percentage changes in volume comparing injured with contralateral thigh volume was not significantly different between graft type groups.

**Conclusions:**

No significant side-to-side differences were found in individual thigh compartments or total volume after allograft ACLR. BPTB ACLR has a modest effect (∼3%) on long-term total thigh muscle volume.

**Level of Evidence:**

Level III, retrospective cohort study.

Anterior cruciate ligament (ACL) injuries are among the most prevalent injuries in athletes, with an estimated incidence of 68.6 per 100,000 person-years.^1^, ^2^, ^3^ Left untreated, ACL injuries can lead to substantial functional impairment, tibiofemoral instability, and accelerated degenerative joint disease, especially in active populations.^4^ Anterior cruciate ligament reconstruction (ACLR) has consistently shown favorable outcomes, including reduced rates of subsequent knee surgeries, decreased incidence of meniscal and cartilage injuries, and improved long-term functional status.^4^^,^^5^ However, knee flexion and extension strength may be significantly decreased postoperatively.^6^

Various graft options are available for ACLR, each with advantages and potential drawbacks. Autografts, such as hamstring tendon (HS) and bone−patellar tendon−bone (BPTB) grafts, are commonly used because of their lower failure rates, which is particularly advantageous in highly active populations. Allografts offer the benefit of reduced donor-site morbidity but may be associated with greater rates of graft laxity and failure, particularly in younger, more active populations.^7^, ^8^, ^9^, ^10^, ^11^, ^12^, ^13^, ^14^, ^15^, ^16^, ^17^ Quadriceps atrophy measured via ultrasound has been shown to be correlated with patient-reported functional outcomes, and although previous studies have investigated factors influencing ACLR outcomes, including graft selection and its effects on muscle strength and function, the impact of alternative graft choices, such as allografts and BPTB autografts, on thigh muscle volume recovery remains unclear.^1^^,^^5^^,^^18^

The purpose of this study is to evaluate thigh muscle volume differences in patients undergoing ACLR with either allograft tissue or BPTB autografts using 3-dimensional (3D) magnetic resonance imaging (MRI) modeling. We hypothesized that anterior compartment muscle changes would persist even 5 years or more after BPTB ACLR, whereas no such changes would be observed with the use of allograft.

## Methods

### Patient Selection

All patients who underwent ACLR with tibialis anterior allografts and ipsilateral BPTB autograft between December 2014 and December 2018 were identified from a single academic center. Inclusion criteria were age 18 to 45 years and a body mass index <30 at the time of surgery. Consecutive patients were screened in sequential fashion after retrospective chart review and were then contacted to ensure that there were no subsequent knee surgeries of the ipsilateral or contralateral side, with enrollment performed on a rolling basis. Those subjects with any knee surgery after the indexed surgery, multiligamentous knee injuries, laxity observed on physical examination postoperatively, and those with any surgical treatment of meniscus (repair or meniscectomy) or cartilage were excluded.^17^ A total of 20 subjects (10 per group) were included. Patients meeting inclusion criteria were then prospectively contacted, consented, and enrolled for follow-up MRI.

### Indication for Surgery and Surgical Technique

All patients presented with symptomatic instability attributable to an ACL tear, confirmed clinically and by advanced imaging. Surgical intervention was indicated for patients who desired to return to recreational or competitive sports or reported ongoing difficulty with pivoting or cutting activities. All reconstructions were performed arthroscopically. Standard anteromedial portals were used to visualize the ACL footprint. Femoral tunnel placement was performed via an anteromedial tunnel drilling technique; the tibial tunnel was prepared using a transtibial approach. Grafts (tibialis anterior allograft or ipsilateral BPTB autograft) were selected on the basis of patient preference, graft availability, and surgeon discretion. Fixation was accomplished with interference screws on both the femoral and tibial sides. A standardized postoperative rehabilitation protocol was used for both graft types.

### Digital Reconstruction of MRI and Segmentation Procedures

For each patient, MRI scans of both the injured and contralateral thighs, spanning from the lesser trochanter to the superior tibial tubercle, were acquired using a Siemens MAGNETOM Vida 3T scanner with voxel dimensions 1.5 mm × 1.5 mm × 3.0 mm. The Digital Imaging and Communications in Medicine files were extracted from the Picture Archiving and Communication System (PACS, IntelliSpace Radiology Enterprise 4.5; Philips North America Corporation) and imported into an open-source, image computing platform (3D Slicer version 5.6.2, The Slicer Community, https://www.slicer.org) for analysis.^19^^,^^20^ T1-weighted images in axial, sagittal, and coronal planes were used for segmentation.

**Fig 1:**
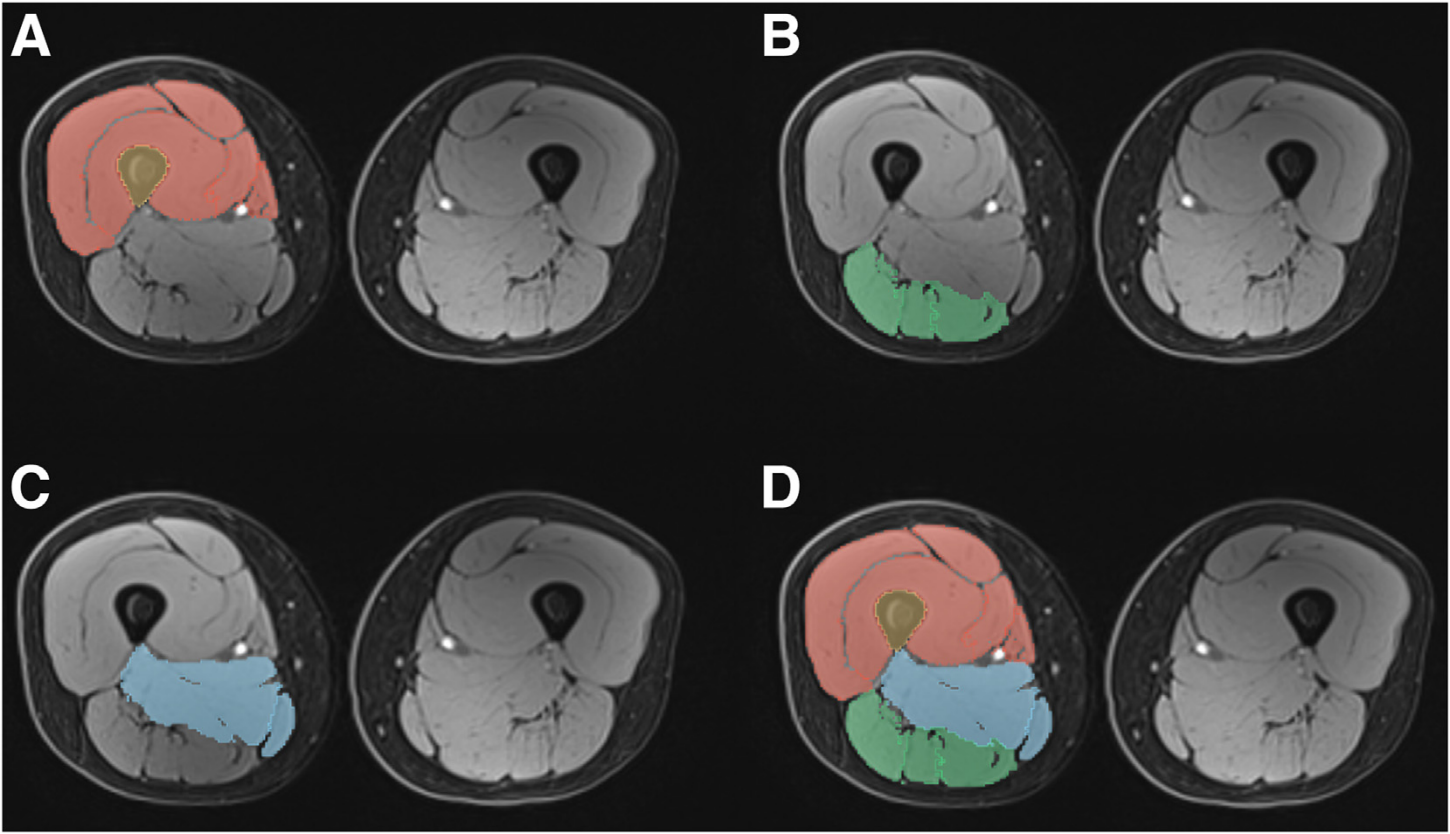
Axial segmentations of thigh muscle compartments. (A) The anterior compartment (red). (B)T posterior compartment (green). (C) The medial compartment (blue). (D) All 3 segmentations overlaid on a single axial slice of one thigh for composite visualization.

**Fig 2:**
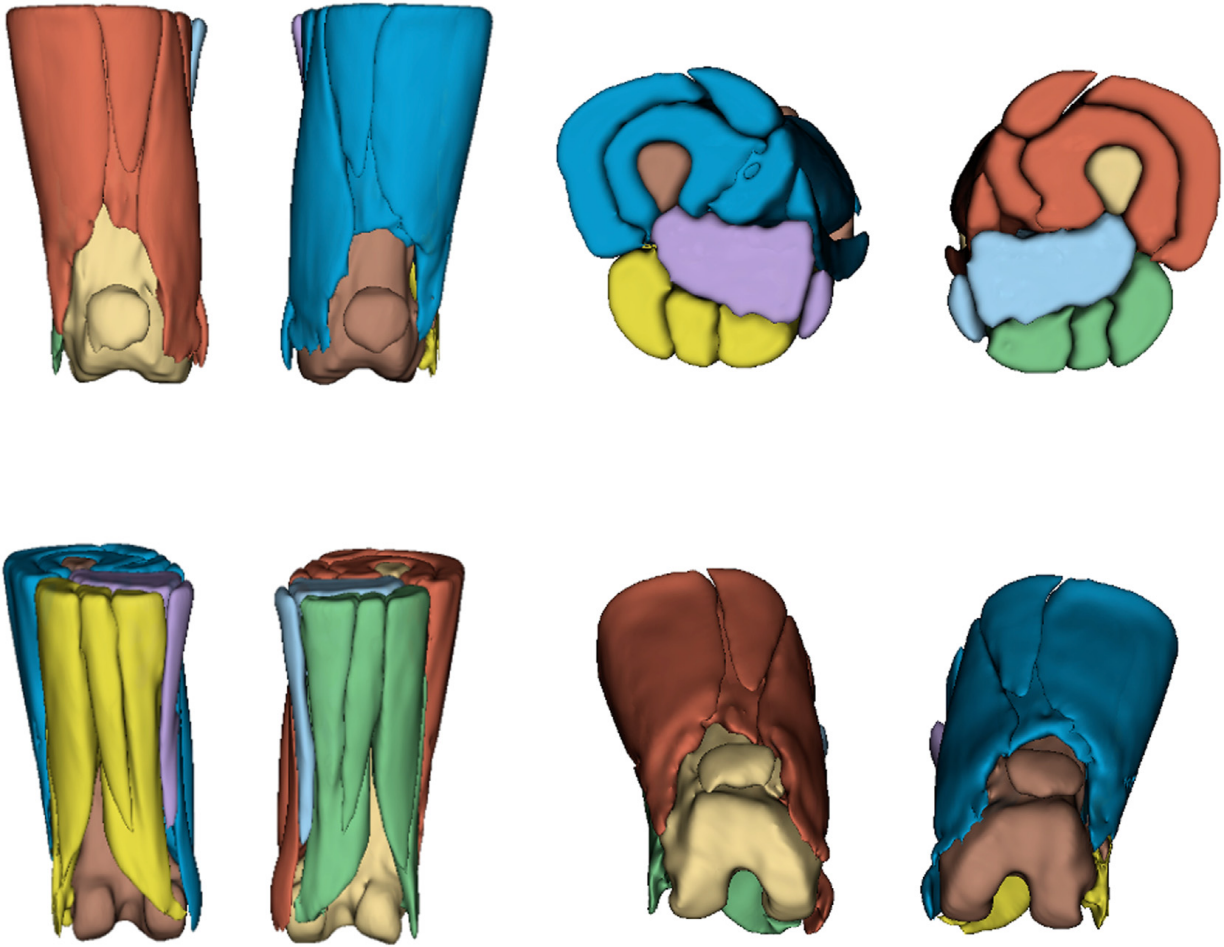
Views of 3-dimensional rendering of thigh muscle compartments.

Within 3D Slicer, the “Segment Editor” module was employed to manually delineate the anterior, posterior, and medial thigh compartments, as well as skin, subcutaneous fat, and neurovascular structures as described in previous literature (Fig 1).^21^ First, the thigh muscle compartment borders were identified manually and outlined starting with the most superior axial plane. Moving distally, the same muscle compartment borders were serially identified and outlined in a sequential fashion. Bone, skin, subcutaneous fat, and neurovascular structures were also segmented in a serial fashion simultaneously. Semiautomated tools were used to enhance segmentation accuracy. The same protocol was then applied to the contralateral thigh in a similar fashion. To address discrepancies in proximal visualization between limbs, a standardized adjustment was implemented: a line was drawn from the posterior aspect of the trochlear notch along the posterior femur to the maximum visible distance on both thighs; in all instances, 3D rendering of the longer limb was adjusted to match the visualization extent of the shorter limb. Subsequently, the delineated contours were used to reconstruct 3D models of each thigh muscle compartment, and muscle volumes were quantified using the corresponding statistical modules, which provides metrics such as voxel count and volume in cubic millimeters and centimeters (Fig 2). This methodology ensured precise and consistent segmentation, facilitating accurate volumetric analysis of thigh muscle. To assess intraobserver reproducibility in the segmentation and measurement process, 2 orthopaedic surgery residents (N.M, R.M.R.) performed these steps on 10 patients independently. The intraclass correlation coefficient was calculated to evaluate interobserver reproducibility.

### Data Collection and Statistical Analysis

Patient information was collected from the electronic medical record (Epic; Epic Systems), including the patient’s age, sex, and body mass index (BMI). Enrollment was completed when 10 consenting patients completed MRI scans in each graft type group, consistent with previously reported methodologies using 3D-reconstructed MRI scan in patients who underwent ACLR.^17^ Volumetric and demographic data were collected and exported to SPSS (version 27; IBM) for statistical analysis. χ^2^ tests were conducted to assess differences in sex distribution and operative laterality between the allograft and BPTB autograft groups. A Shapiro-Wilk test was conducted to test normality among each graft type group and further, each muscle compartment, in an individual fashion. For continuous variables, independent Student *t* tests were used to compare the means of age at the time of ACLR, age at MRI acquisition, mean follow-up time from ACL surgery to MRI, and BMI between the 2 graft groups. Prespecified hypothesis tests were performed on a comparison bases with 2-sided alpha significance set at 0.05.

For muscle volume analysis, thigh muscle volumes of the injured limb’s compartments, as well as the total thigh musculature volume, were compared with the corresponding compartments of the contralateral limb. Paired Student *t* tests were used within each graft group (allograft and BPTB autograft) to assess differences in muscle compartment volumes between the injured and contralateral thighs. To evaluate relative muscle volume changes between graft types, we calculated the proportion of volume change for each compartment by dividing the injured limb’s muscle compartment volume by that of the contralateral compartment ($\frac{injured\;muscle\;compartment\;volume}{contralateral\;muscle\;compartment\;volume}$). The mean proportions of volume change for each muscle compartment were then compared between the allograft and BPTB autograft groups to identify any significant differences. Wilcoxon signed-rank tests were also performed as a nonparametric sensitivity analysis to confirm the robustness of findings from the paired *t* tests. Comparisons of relative volume change between graft groups (injured/contralateral ratios) were conducted using independent *t* tests. Statistical significance was set at a 2-sided alpha level of 0.05.

## Results

### Demographic and Baseline Characteristics

Between 2014 and 2018, a total of 173 BPTB autograft and 329 allograft ACLRs were performed. A total of 5 surgeons performed ACLRs across the 20 included patients. The determination of graft choice was a shared decision with the surgeon and patient. From the database, patients were identified and contacted in sequential order for enrollment. The allograft group was significantly older than the BPTB autograft group at the time of surgery (34.6 years vs 23.4 years; *P* < .001) and at the time of study MRI (40.9 years vs 29.7 years; *P* < .001). No significant differences were observed between the groups in terms of mean follow-up time from ACLR to study MRI, sex distribution, BMI, or operative laterality (Table 1). Interobserver reproducibility for 3D volumetric measurements of thigh muscle compartments was rated as “good,” with an intraclass correlation coefficient of 0.753 across raters.^22^ To confirm the robustness of our findings, Wilcoxon signed-rank tests were performed within each graft group. In the BPTB group, there were statistically significant reductions in muscle volume in the anterior compartment (Z = –2.293, *P* = .022) and adductor/medial compartment (Z = –2.090, *P* = .037) when we compared the injured limb with the contralateral limb. No significant differences were found in the posterior compartment (Z = –0.866, *P* = .386). In contrast, the allograft group did not show significant volume differences in any compartment: anterior (Z = –1.478, *P* = .139), medial (Z = –0.357, *P* = .721), or posterior (Z = –1.274, *P* = .203).

**Table 1:** Demographics of Allograft and BPTB ACLR Study Participants

Variable	Allograft Mean (SD)	BPTB Mean (SD)	*P* Value
Age at ACL reconstruction, yr	34.59 (5.17)	23.44 (4.38)	<.001
Age at study MRI, yr	40.85 (4.86)	29.72 (5.03)	<.001
Mean follow-up time, yr	6.27 (1.14)	6.28 (1.33)	.492
Male, n (%)	4 (40%)	9 (90%)	.057
BMI	23.42 (3.29)	23.50 (2.55)	.495
Operative laterality, left, n (%)	6 (60%)	5 (50%)	.712

NOTE. Values are mean (SD) unless otherwise specified.

ACL, anterior cruciate ligament; ACLR, anterior cruciate ligament reconstruction; BMI, body mass index; BPTB, bone−patellar tendon−bone; MRI, magnetic resonance imaging; SD, standard deviation.

### Thigh Muscle Volume Comparison Between Injured and Contralateral Limbs

The mean volumetric measurements of thigh muscle compartments within the allograft group comparing injured and contralateral limbs showed no significant differences (Table 2). In contrast, the BPTB autograft group showed a significant reduction in total thigh muscle volume in the injured limb compared with the contralateral limb (3,013.13 cm^3^ vs 3,116.54 cm^3^, respectively; *P* = .013). However, individual compartments in the BPTB group did not show statistically significant differences between the injured and contralateral thigh. The anterior compartment volumes were 1,558.84 cm^3^ in the injured limb and 1,548.72 cm^3^ in the contralateral limb (*P* = .676). For the medial compartment, the volumes were 329.37 cm^3^ in the injured limb and 331.85 cm^3^ in the contralateral limb (*P* = .914). Finally, the posterior compartment volumes were 927.61 cm^3^ in the injured limb and 929.26 cm^3^ in the contralateral limb (*P* = .959) (Table 3).

**Table 2:** Volumetric Measurements of Allograft ACLR Thigh Musculature Volume (Mean, SD) and Statistical Comparison Between the Operative and Nonoperative Extremity

Allograft	Mean Operative Muscle Volume, cm^3^	Mean Nonoperative Muscle Volume, cm^3^	Mean Muscle Difference %, (Injured/Contralateral) (SD)	*P* Value
Total thigh	1,977.12 (896.7)	1,990.80 (910.3)	99.4 (3.4)	.479
Anterior compartment	1,144.33 (535.2)	1,168.29 (542.2)	98.0 (3.6)	.102
Medial compartment	234.58 (138.1)	237.55 (158.6)	98.7 (17.4)	.820
Posterior compartment	598.50 (241.9)	584.97 (222.7)	101.3 (6.0)	.234

ACLR, anterior cruciate ligament reconstruction; SD, standard deviation.

**Table 3:** Volumetric Measurements of BPTB ACLR Thigh Musculature Volume (Mean, SD) and Statistical Comparison Between Operative and Nonoperative Extremity

BPTB	Mean Operative Muscle Volume, cm^3^	Mean Nonoperative Muscle Volume, cm^3^	Mean Muscle Difference %, (Injured/Contralateral) (SD)	*P* Value
Total thigh	3,013.13 (1183.4)	3,116.54 (1217.0)	97.0 (3.8)	.013∗
Anterior compartment	1,558.84 (626.3)	1,548.72 (626.3)	100.7 (5.7)	.676
Medial compartment	329.37 (179.8)	331.85 (145.7)	97.4 (19.0)	.914
Posterior compartment	927.61 (390.4)	929.26 (349.9)	99.0 (10.1)	.959

ACLR, anterior cruciate ligament reconstruction; BPTB, bone−patellar tendon−bone; SD, standard deviation.

∗ Indicates statistical significance (*P* < .05).

### Comparison of Muscle Volume Change Proportions Between Graft Types

Although the BPTB group showed a trend toward greater reduction in total thigh volume relative to the contralateral limb compared with the allograft group, this difference was not statistically significant (*P* = .315). No significant differences were observed between graft groups for individual muscle compartments when we compared proportional volume changes (Table 4).

**Table 4:** Comparison of Mean Volumetric Change Percentages (Injured/Contralateral) Between BPTB and Allograft Groups

Variable	Allograft Δ% (SD)	BPTB Δ% (SD)	*P* Value
Total thigh volume	99.4 (3.4)	97.0 (3.8)	.315
Anterior muscle volume	98.0 (3.6)	100.7 (5.7)	.196
Medial muscle volume	98.7 (17.4)	97.4 (19.0)	.457
Posterior muscle volume	101.3 (6.0)	99.0 (10.1)	.563

BPTB, bone−patellar tendon−bone; SD, standard deviation.

## Discussion

The primary finding of this study was that we identified a statistically significant 2.4% reduction in total thigh muscle volume in the ipsilateral (injured) limb compared with the contralateral (uninjured) limb at a minimum of 5 years post-BPTB autograft ACLR. Notably, this reduction in overall muscle volume was not observed in the individual muscle compartments of the anterior, medial, or posterior thigh in the BPTB group, suggesting that the volumetric atrophy is distributed uniformly across the thigh musculature rather than being localized to specific compartments. Thus, our hypothesis regarding anterior compartment persistent atrophy at long-term was rejected. Conversely, patients who underwent ACLR with allografts did not exhibit significant differences in muscle volume between the injured and contralateral limbs, either in total thigh volume or within specific compartments. These findings offer important anatomical insights into the long-term impact of ACLR on thigh muscle volume and raise questions about the relationship between graft type and muscle preservation.

To account for the limited sample size and potential fragility of parametric assumptions, we conducted Wilcoxon signed-rank tests as a nonparametric sensitivity analysis. These tests confirmed statistically significant reductions in anterior and medial thigh muscle volumes in the BPTB autograft group, consistent with the results of the paired *t* tests. In contrast, no compartmental differences were observed in the allograft group across either analysis. The alignment between the parametric and nonparametric tests supports the robustness of our findings despite the relatively small sample size and reinforces the interpretation that the thigh muscle volume decreases after BPTB autograft ACLR.

Although much of the existing literature on ACLR has focused on functional outcomes, return-to-sport rates, graft failure risk factors, and pre- and postoperative strength differences,^7^, ^8^, ^9^, ^10^^,^^23^, ^24^, ^25^, ^26^ relatively little attention has been directed toward the long-term effects of ACLR on thigh muscle volume. This is an important consideration, particularly when selecting graft types tailored to individual athletic and functional demands. For example, quadriceps tendon (QT) autografts often are avoided in high-level alpine skiers to preserve extensor mechanism strength, whereas sprinting athletes may avoid HS autografts because of the risk of knee flexion deficits that are essential for the biomechanics of sprinting.^27^ Persistent donor-site muscle strength deficits have been reported after BPTB, HS, and QT autografts,^28^, ^29^, ^30^, ^31^ emphasizing the need for a deeper understanding of the factors driving these deficits. Although it remains unclear whether the present study’s data revealing muscle loss in the affected leg directly translates to clinically meaningful strength deficits, the findings align with previous reports of donor-site morbidity and suggest that graft choice may influence long-term muscular preservation—an important consideration in optimizing recovery, performance, and joint health in physically active patients.

Our findings suggest that volumetric muscle atrophy in the anterior thigh after BPTB autograft ACLR is minimal, with a mean reduction of approximately 103.41 cm^3^, roughly equivalent to the volume of 3 standard golf balls. Although this muscle volume reduction could have structural significance, it is unlikely to account for persistent strength deficits at long-term follow-up. Instead, such deficits may be attributable to other factors, including neuromuscular adaptations, altered biomechanics, or tendon-related issues.^32^, ^33^, ^34^ In contrast, the absence of volumetric muscle reductions in patients with allografts highlights the potential advantage of avoiding donor-site morbidity altogether, although the functional implications of this finding remain uncertain. Further research correlating muscle volume with functional testing is needed to determine whether these anatomical differences translate to clinically meaningful outcomes. Currently, cut-off values and minimal clinically important differences for thigh muscle volume loss after ACLR remain poorly understood, underscoring the need for future studies to define thresholds that correlate with functional outcomes.

Previous studies have extensively documented the impact of HS autografts on thigh musculature, particularly persistent atrophy in the semitendinosus and gracilis muscles.^17^^,^^23^^,^^35^ For instance, Snow et al.^17^ found significant long-term atrophy in these muscles after ACLR with HS autografts, with a reported 40% to 50% reduction in gracilis and semitendinosus muscle volume, partially offset by hypertrophy of the biceps femoris long head. Although the present study did not reveal comparable atrophy patterns in BPTB autografts, this discrepancy could reflect differences in donor-site anatomy and biomechanical demands between the graft types. By evaluating total and compartmental thigh muscle volume in BPTB and allograft groups, our study addresses a critical gap in the literature, providing a more comprehensive understanding of the long-term anatomical effects of these graft options. Future studies should assess individual muscles within each compartment to detect more localized patterns of atrophy or compensatory hypertrophy that may have been missed in our analysis. For example, specific muscles within the anterior compartment, such as the vastus medialis or rectus femoris, may exhibit distinct responses to graft harvest that could mirror the atrophy patterns observed by Snow et al. in HS autografts. Such studies would enhance our understanding of the anatomical changes associated with BPTB autografts and their potential implications for strength and function.

### Limitations

This study is subject to several limitations. First, the relatively small sample size may reduce the statistical power and may result in a type II error. There were substantial financial limitations, such as the cost of bilateral MRI, that impacted our ability to include more subjects. In addition, our cross-sectional design limits the ability to track muscle volume changes over time; a prospective, longitudinal approach could better elucidate the trajectory of muscle recovery and distinguish between transient atrophy and persistent loss. The absence of preoperative baseline measurements also restricts our ability to differentiate between injury-related atrophy and changes attributable to the reconstruction procedure. Furthermore, the lack of comparison groups using alternative graft types, such as HS and QT autografts, limits the scope of our findings. Another limitation of our study is the sex differences in our 2 graft groups (40% male in allografts, 90% male in BPTB). Although this difference was not statistically significant, it is notable. In addition, the mean patient age in each group was significantly different and is a limitation of our study enrollment design. Furthermore, because of the limited number of patients included in this study, disaggregating the available data by sex is not possible. In this study, only muscle volume was evaluated without access of muscle strength or association with clinical outcome. For clinical relevance, the muscle strength or association with clinical outcome should be evaluated. Finally, assessing thigh muscle compartments rather than individual muscles may have obscured localized patterns of atrophy or hypertrophy within specific muscles.

## Conclusions

No significant side-to-side differences were found in individual thigh compartments or total volume after allograft ACLR. BPTB ACLR has a modest effect (∼3%) on long-term total thigh muscle volume.

## Disclosures

The authors declare the following financial interests/personal relationships which may be considered as potential competing interests: S.K.A. reports consulting or advisory and travel reimbursement from Stryker. J.J.E. reports consulting or advisory and travel reimbursement from Medical Devices Business Services; travel reimbursement from DePuy Synthes; and reviewer for *Arthroscopy*. J.F. reports travel reimbursement from Globus Medical and Stryker. P.G. reports travel reimbursement from Smith & Nephew. T.G.M. reports consulting or advisory, speaking and lecture fees, and travel reimbursement from Arthrex. All other authors (C.D.R.H., N.M., R.M.R.) declare that they have no known competing financial interests or personal relationships that could have appeared to influence the work reported in this paper.
